# Screen Mesh Size for Exclusion of *Diaphorina citri* (Hemiptera: Liviidae) in Citrus Production

**DOI:** 10.1093/jee/toaa095

**Published:** 2020-05-19

**Authors:** Timothy A Ebert, Laura Waldo, Daniel Stanton, Arnold W Schumann

**Affiliations:** 1 Department of Entomology and Nematology, Citrus Research and Education Center, Institute of Food and Agricultural Sciences, University of Florida, 700 Experiment Station Rd. Lake Alfred, Florida; 2 Department of Soil and Water Science, Citrus Research and Education Center, Institute of Food and Agricultural Sciences, University of Florida, 700 Experiment Station Rd. Lake Alfred, Florida; 3 Microscopy Facility, Citrus Research and Education Center, Institute of Food and Agricultural Sciences, University of Florida, 700 Experiment Station Rd. Lake Alfred, Florida

**Keywords:** *C*Las, HLB, greening, Liberibacter, pest management

## Abstract

Huanglongbing is a citrus disease that reduces yield, crop quality, and eventually causes tree mortality. The putative causal agent, *Candidatus* Liberibacter asiaticus (Rhizobiales: Rhizobiaceae), is vectored by the Asian citrus psyllid, *Diaphorina citri* Kuwayama. Disease management is largely through vector control, but the insect is developing pesticide resistance. A nonchemical approach to vector management is to grow citrus under screen cages either as bags over individual trees or enclosures spanning many acres. The enclosing screen reduces wind, alters temperature relative to ambient, and excludes a variety of pests that are too large to pass through the screen. Here we evaluated the potential of six screens to exclude *D. citri*. We conclude that screens with rectangular openings need to limit the short side to no more than 384.3 µm with a SD of 36.9 µm (40 mesh) to prevent psyllids from passing through the screen. The long side can be at least 833 µm, but the efficacy of screens exceeding this value should be tested before using in the field.

*Diaphorina citri* Kuwayama transmits a phloem-limited alpha proteobacterium, *Candidatus* Liberibacter asiaticus (Rhizobiales: Rhizobiaceae). *Candidatus* Liberibacter asiaticus (*C*Las) is the putative causal agent for Huanglongbing in citrus. Since its introduction into Florida in 2005 (Halbert 2005), citrus yields have declined by 68% from 2004 to 2016 (https://www.nass.usda.gov/Statistics_by_State/Florida). *Candidatus* Liberibacter asiaticus is a global problem (Bove 2006), and others may face a similar fate as *C*Las invades new citrus-growing regions as it has done in California and Texas (Kumagai et al. 2013, Kunta et al. 2014).

Disease management involves vector management, removing infected trees, and disease-free nursery production (Grafton-Cardwell et al. 2013). However, *D. citri* has developed insecticide resistance to carbamates (methomyl), organophosphates (chlorpyrifos, dimethoate, malathion), pyrethroids (bifenthrin, fenpropathrin, lambda-cyhalothrin), neonicotinoids (acetamiprid, dinotefuran, imidacloprid, nitenpyram, thiamethoxam), and an uncoupler of oxidative phosphorylation through disruption of the proton gradient (chlorfenapyr) (Tiwari et al. 2011, Garcia-Mendez et al. 2016, Naeem and Freed 2018, Pardo et al. 2018, Tian et al. 2018). Within one population, resistance may develop to these or other pesticides, but the effectiveness of resistance management strategies depends on the specific insecticide (Chen et al. 2017). Disease management through vector control will fail if resistance continues to develop.

Two nonchemical approaches to psyllid management are used in Florida. Citrus Under Protective Screen (CUPS) is where the entire grove is enclosed under a screen. The alternative encloses individual trees in a screen bag during the first 2 yr of vegetative growth (https://thetreedefender.com/). Screened enclosures are used extensively in commercial agriculture (Tanny 2013, Chouinard et al. 2016, Nordey et al. 2017, Fernandez et al. 2018, Mahmood et al. 2018, Mupambi et al. 2018, Ingwell and Kaplan 2019, Merfield et al. 2019).

Determining the best opening size is important when selecting screen. Smaller openings not only protect against smaller pests, but also reduce gas exchange, increase humidity and temperature, and reduce wind speeds (Teitela and Wenger 2014, Rathee et al. 2018). These consequences can be beneficial or detrimental depending on the response of nonexcluded pests to the altered environment. In CUPS, the screen is a pesticide drift management barrier because the sprayer is inside the enclosure (Fritz et al. 2010). A detailed introduction to this practice was reviewed recently (Manja and Aoun 2019). For additional information on CUPS in Florida, see http://www.makecitrusgreatagain.com/CUPS.htm (accessed 3 March 2020).

To identify the best screen, we characterized several screens using scanning electron microscopy (SEM) images and determined the risk of psyllids penetrating the screen using a bioassay chamber. We measured insect height and width under the assumption that psyllid size influences the probability of screen penetration. We then estimated the probability that a psyllid could pass through the screen.

## Materials and Methods

**Fig. 1.**
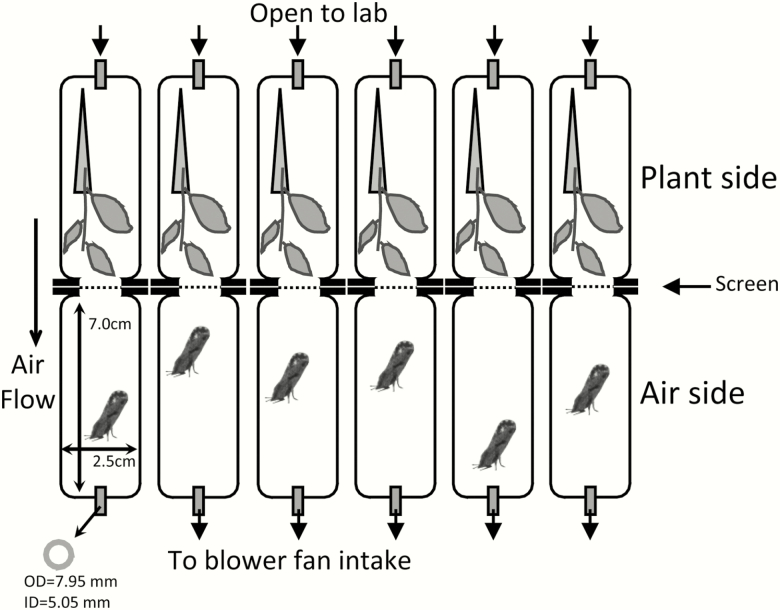
Schematic of the six test chambers for measuring the ability of *Diaphorina citri* to pass through screens of different mesh sizes. All insects start on the air side of the test arena and must pass through a screen to reach food and water. The psyllid images are about 10× life size relative to the scale of the drawing. Each arena would have multiple insects for each run of the experiment. See Tables 2 and 3 for sample sizes.

To measure penetration, we used six arenas wherein laboratory air was pulled over new citrus flush (apical meristem plus 2 or 3 leaves < 30% fully expanded), through a test screen, and over recently collected adult psyllids (Fig. 1). Each test lasted 48 h. Adult psyllids unable to penetrate the screen died from desiccation. An arena consisted of two nine-dram styrene vials (model 8909, Bioquip.com, Rancho Dominguez, CA). Holes were cut into the center of each lid, and the lids stapled together. The hole was covered with a screen on the side facing the vial with adult psyllids. Flush was maintained for 48 h by placing the stem in a water filled 1.5-ml centrifuge tube (model 05-408-129, Thermofisher.com). The bottom of each styrene vial had a hole that was plugged with PVC tubing (model 714422, Homedepot.com). Glass wool was loosely packed into the end of the tube to prevent psyllids from escaping. The side of the arena with food was open to ambient laboratory air. The side of the arena with psyllids went to a manifold consisting of a schedule 40 PVC male adapter (model D2466, Lasco Fittings Inc., Brownsville, TN) with six holes drilled into the sides below the threaded end. An end cap (model 447020, Lasco Fittings Inc.) rested over the male fitting to restrict airflow. The male adapter was glued over the intake of a blower fan (model COM-11270, Sparkfun Electronics Niwot CO USA, 12 VDC 0.9 amp 10 Watt). A power supply delivered 6VDC (model CS13003X III, CircuitSpecialists.com). The packing of the glass wool was adjusted to equalize the flow rate to 50 ft/min at the intake tube as measured with an Alnor velometer Jr. (model 8100, TSI Inc., Shoreview, MN).

**Table 2.** An estimated percentage of *Diaphorina citri* able to pass through screens with different sized openings and the 95% confidence interval for this estimate

		Total	NumberPassed	Percentage	95% confidence interval		
Screen	Vials	Tested		Passed	Low	High	Fraction
PME066	11	184	137	74.5	67.8	80.4	2.7
PME096	49	1488	113	7.6	6.3	9.0	1.6
PolySack25	12	275	3	1.1	0.3	2.9	1.4
PME108	12	311	1	0.3	0.0	1.5	1.5
PME1610	12	334	0	0.0	0.0	1.1	0.8
PolySack40	12	273	0	0.0	0.0	1.4	0.8

The number of times the experiment was run (vials) and the total number of psyllids tested are included with the total number of psyllids that passed through the screen. For this table, insect is the replicate in calculating the estimate and confidence intervals. Fraction is the ratio of the smallest screen dimension divided by the smallest psyllid dimension.

**Table 3.** Testing whether the size of the psyllids (see Fig. 2) is different between the air or leaf side of the test chamber (see Fig. 1)

Screen	Side	Insects	Height ± SD (mm)	*P*-value	Width ± SD (mm)	*P*-value
PME066	Air	6	0.63 ± 0.05	0.2424	0.61 ± 0.05	0.9114
	Leaf	87	0.65 ± 0.04		0.60 ± 0.03	
PME096	Air	160	0.64 ± 0.04	0.2928	0.61 ± 0.03	<0.0001
	Leaf	45	0.64 ± 0.05		0.59 ± 0.03	

Insects are the number of insects in total in each treatment category. The *P*-values are for the difference between the measurement for insects on the air side versus insects on the leaf side.

We counted the number of psyllids that moved through the screen and the number that failed to do so. The screens in order of decreasing opening size were as follows: PME066 (ULMA S. Coop., Oñati, Gipuzkoa, Spain); PME096 (ULMA S. Coop.); PME108 (ULMA S. Coop.); Polysack25 (Green.tek, Janesville, WI), HDPE 25 mesh (model WEM2525040000, Ginegar Plastic Products Ltd., Kibbutz Ginegar, Israel); PME1610 (ULMA S. Coop.); Polysack40 (model WEM4025040000, Green.tek, HDPE 40 mesh, Ginegar Plastic Products Ltd.).

**Fig. 2.**
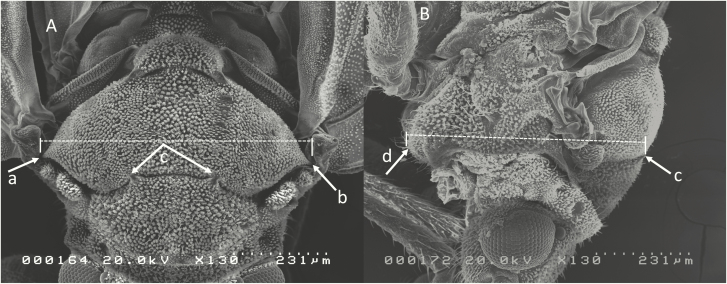
SEM image of external morphology of *Diaphorina citri* showing the measurement of width (A) and height (B). Outermost arrows (2Aa and 2Ab) are the morphological features defining width. The arrows marked ‘c’ in (A) indicate the same morphological feature as arrow ‘c’ in (B). Arrow ‘d’ is determined by the curvature of the sclerite. Measurements were used as an estimate of size in determining the insect’s ability to pass through screens of different hole sizes.

Adult psyllids were collected from a *C*Las-free laboratory colony fed curry leaf [*Murraya koenigii* (L.) Spreng (Sapindales: Rutaceae)] and from a variety of sweet orange, tangerine, and grapefruit trees grown in conventional citrus groves at the University of Florida IFAS Citrus Research and Education Center (Lake Alfred, FL). Many grove psyllids were likely infected with *C*Las, but this was not confirmed. Psyllid size was measured as the width of the metathoracic tergite, as shown in Fig. 2A. The height of the insect was measured from the mesothoracic sternum to a point along the suture between the meso- and metathoracic tergites, as marked in Fig. 2A and B.

Adult psyllids were sampled arbitrarily from the grove or growth chamber. The sample sizes for each treatment are provided in tables within the results section. There was no effort to control the ratio of male to female, psyllid age, or other traits. The sex ratio for this psyllid is often close to 50:50 (Tsai and Liu 2000, Hall 2018), so an arbitrary sample of several hundred should have both males and females. It was assumed that the primary factor influencing an insect’s ability to pass through the screen was a function of insect size and mesh size. SEM images showed rectangular openings and initial experiments had not considered this as a factor. A final experiment was done where the long axis of the openings in PME096 screen was oriented vertically or horizontally with respect to gravity. These data were subsequently combined with the rest of the data.

Scanning electron microscopy: Adult psyllids were frozen, then dehydrated in an ethanol series (25, 50, 75, and 100%) at room temperature. Screens and psyllids were dried overnight at 40°C. Samples were placed on carbon adhesive tabs on 12-mm aluminum stubs and sputter-coated with a gold/palladium mixture using a Ladd 30802 (Ladd Research Industries Inc., Williston, VT). Samples were photographed using a Hitachi S-4000 SEM (Hitachi High Technologies, Tokyo, Japan). SEM was used on screens to get accurate measurements of opening size and thread diameter and show defects in the threads. SEM of the insect was used to document how the insects were measured.

Screens were characterized using means and SD for opening size and fiber diameters. The proportion of psyllids that can pass through each screen was estimated using the binGroup package (Zhang et al. 2018) in R (version 3.5.1: R Core Team 2018) running in RStudio (version 1.1.456). ANOVA was run in SAS (version 9.4 TS 1M3) in SAS Enterprise Guide (version 7.15 HF6 [7.100.5.6165]) with side (air vs leaf) as the independent variable and height or width as the dependent variable to test if orientation relative to gravity made a difference. The binGroup package was used to calculate the proportion of adults that pass through the screen and a 95% confidence interval for each of 15 runs. A regression analysis was run in R (using lm) to estimate the screen size and 95% prediction limits where no psyllids could pass using the shortest opening dimension as the independent variable and the proportion of psyllids passing the screen as the dependent variable. The two screens where no psyllids penetrated the screen were excluded from this analysis.

## Results

**Fig. 3.**
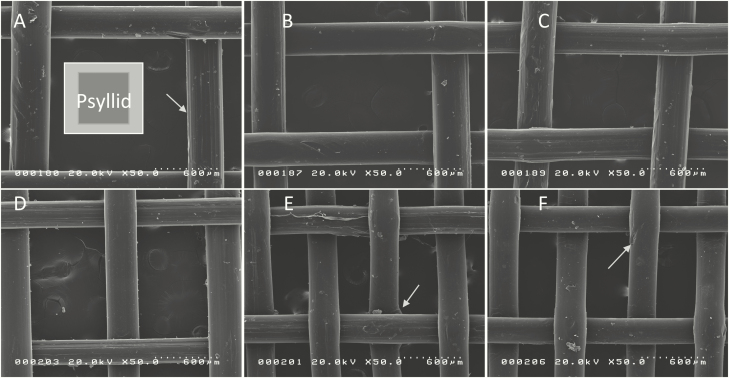
SEM image of the six screens: (A) PME066, (B) PME096, (C) PME108, (D) Polysack25, (E) PME1610, (F) Polysack40. Arrows point out small defects in screen manufacture. The square marked ‘psyllid’ is the largest and smallest measured dimensions out of 336 males and 337 females.

The holes in all screens were rectangular (Table 1). The fibers running the long dimension were finer than fibers on the short dimension. All screens showed shallow grooves in fibers (Fig. 3A), flaring of fibers at intersections (Fig. 3E), and gouges in the fibers (Fig. 3F). Such features might be large enough to allow the tarsal claws of psyllids to grip the fibers.

**Table 1.** Characterization of six screens based on hole size and fiber diameters

Screen	Advertised	Hole size (µm)		Fiber diameter (µm)		Vertical	Horizontal	
Name	Mesh size	Short side	Long side	Short side	Long side	Mesh	Mesh	Porosity^*a*^
PME066	17	1,303.0 ± 11.4	1,383.8 ± 37.5	370.1 ± 1.5	317.9 ± 7.7	15.2 ± 0.1	14.9 ± 0.4	0.633 ± 0.005
PME096	20	788.9 ± 64.6	1,438.0 ± 20.9	347.0 ± 10.1	314.1 ± 7.4	23.1 ± 1.2	14.5 ± 0.2	0.570 ± 0.018
Polysack25	25	675.7 ± 21.8	1,094.0 ± 8.0	243.2 ± 13.4	321.8 ± 15.3	25.5 ± 0.8	17.9 ± 0.2	0.568 ± 0.010
PME108	30	732.9 ± 23.8	993.6 ± 15.3	352.3 ± 8.8	321.8 ± 9.8	24.1 ± 0.5	19.3 ± 0.1	0.510 ± 0.009
PME1610	40	376.1 ± 25.6	801.7 ± 36.5	279.5 ± 8.3	276.9 ± 11.2	38.7 ± 0.4	23.6 ± 0.9	0.426 ± 0.009
Polysack40	40	384.3 ± 36.9	833.3 ± 26.5	258.0 ± 1.9	282.3 ± 6.6	39.6 ± 0.7	22.8 ± 0.6	0.450 ± 0.007

The opening size can be compared with the size of Diaphorina *citri*, the smallest of which measured 487.5 µm wide and 487.5 µm high. Also, a comparison of advertised versus measured mesh size. Screens are sold based on mesh size. The estimated mesh size was based on adding fiber width and hole dimensions and the number of times that sum fits into 2.54 cm.

^*a*^Porosity is the fraction of screen that is open divided by the total area covered.

Adult psyllids penetrated screen PME066 with 74.5% (*n* = 184) able to pass through the screen. Only 7.6% (*n* = 1,488) of psyllids passed PME096. No psyllid passed the screens with the smallest openings PME1610 and Polysack40 (*n* = 334 and *n* = 273, respectively; Table 2). While no psyllids penetrated these screens, the upper 95% confidence interval for this was less than 1.5%. Even a sample size hundreds of times larger would not be enough to guarantee that no psyllid could ever penetrate the screen.

Both the PME066 and PME096 screens allowed some psyllids to pass but also excluded some. For these two screens, we measured the height and width of psyllids (Table 3). Psyllids were slightly taller than wide. Because the exoskeleton makes some movements more difficult than others, the chance of penetration may change depending on the dexterity of the psyllid and how its body is oriented relative to the rectangular opening in the screen. For PME066, there was no difference in either height or width related to whether the psyllid was on the side with the plant or not. For PME096, there was no effect of height, but there was a significant difference for width where smaller insects were more likely to get through the screen (df = 1,202, *F* = 20.64, *P* > *F* < 0.0001). However, width explained little of the variability in the significant model (*r*^2^ = 0.09).

A regression analysis using data from screens where some psyllids passed through the screen was significant (df = 1,2, *F* = 190.9, *P* > *F* = 0.005) with the equation for the proportion of psyllids passed = −0.8728 (0.0815) + 0.00124 (0.00009) × opening size. The lower 95% prediction for the short side dimension that would be ‘psyllid proof’ was 495.5 µm. This was at least 110 µm larger than the short side of the screens that did not let psyllids pass.

Insects orient to their environment and gravity is an easily detected cue. On a vertical screen, psyllids may respond differently depending on the orientation of the rectangular screen openings relative to the direction of gravity. However, regardless of orientation, the probability that a psyllid penetrated a PME096 screen did not change (Table 4). While we did not standardize our other experiments regarding screen orientation, this result indicates that this detail was unimportant in this experiment.

**Table 4.** The probability of a psyllid passing through a PME096 screen oriented horizontally (long axis at a right angle to gravity) or vertically (long axis oriented with gravity)

	Horizontal orientation			Vertical orientation		
		95% confidence interval			95% confidence interval	
	Estimated	Lower	Upper	Estimated	Lower	Upper
Mean	0.061	0.022	0.170	0.054	0.022	0.169
Median	0.042	0.002	0.141	0.020	0.001	0.132
SD	0.085	0.050	0.108	0.103	0.063	0.126

The probability of getting through the screen was calculated for each of the 15 vials per treatment with a total of 563 psyllids in horizontal and 530 psyllids in vertical treatments. Values were averaged over the 15 vials. Overlapping 95% confidence intervals indicate no significant difference.

## Discussion

The PME1610 and Polysack40 screens kept out all psyllids. A regression analysis indicated that these screens would keep out all psyllids even if the screen stretches slightly under stress, weathering, and age, or if fibers are slightly out of alignment. However, the efficacy of the screen will be impaired more by distortions in the short dimension. Finally, while tested psyllids came from different hosts there may be even smaller psyllids. However, smaller psyllids have fewer resources to survive the migration that would take them from their current host to the plants under protective screen.

We did not get a useful model predicting the ability of insects to penetrate the screen based on insect size and hole dimensions. This outcome has been reported previously (Bethke and Paine 1991). For several insect species, the holes needed to be less than 1.5 times the size of the insect (Bethke and Paine 1991). Our results showed that a hole size 1.4–1.5 times larger than the smallest measured psyllid allows a few psyllids to pass (0.3–1.1%).

We suggest use of screens with openings under 385 µm (40 mesh) because these exclude *D. citri*. In addition to excluding psyllids, the use of these screens further alters the pest management landscape by excluding larger insects: sharpshooters, stink bugs, weevils, and many lepidopterous pests. Beneficial insects are also excluded which may or may not be a benefit.
